# Pro-inflammatory Vascular Stress in Spontaneously Hypertensive Rats Associated With High Physical Activity Cannot Be Attenuated by Aldosterone Blockade

**DOI:** 10.3389/fcvm.2021.699283

**Published:** 2021-07-26

**Authors:** Rolf Schreckenberg, Annemarie Wolf, Christian Troidl, Sakine Simsekyilmaz, Klaus-Dieter Schlüter

**Affiliations:** ^1^Department of Physiology, Justus-Liebig-University Giessen, Giessen, Germany; ^2^Department of Cardiology, Justus-Liebig-University Giessen, Giessen, Germany; ^3^Department of Pharmacology and Clinical Pharmacology, Heinrich-Heine-University Dusseldorf, Dusseldorf, Germany

**Keywords:** NK-cells, IL-6, pcsk9, neutrophils, PAI-1

## Abstract

The effect of high physical activity, performed as voluntary running wheel exercise, on inflammation and vascular adaptation may differ between normotensive and spontaneously hypertensive rats (SHRs). We investigated the effects of running wheel activity on leukocyte mobilization, neutrophil migration into the vascular wall (aorta), and transcriptional adaptation of the vascular wall and compared and combined the effects of high physical activity with that of pharmacological treatment (aldosterone antagonist spironolactone). At the start of the 6th week of life, before hypertension became established in SHRs, rats were provided with a running wheel over a period of 10-months'. To investigate to what extent training-induced changes may underlie a possible regression, controls were also generated by removal of the running wheel for the last 4 months. Aldosterone blockade was achieved upon oral administration of Spironolactone in the corresponding treatment groups for the last 4 months. The number of circulating blood cells was quantified by FACS analysis of peripheral blood. mRNA expression of selected proteins was quantified by RT-PCR. Histology and confocal laser microscopy were used to monitor cell migration. Although voluntary running wheel exercise reduced the number of circulating neutrophils in normotensive rats, it rather increased it in SHRs. Furthermore, running wheel activity in SHRs but not normotensive rats increased the number of natural killer (NK)-cells. Except of the increased expression of plasminogen activator inhibitor (PAI)-1 and reduction of von Willebrand factor (vWF), running wheel activity exerted a different transcriptional response in the vascular tissue of normotensive and hypertensive rats, i.e., lack of reduction of the pro-inflammatory IL-6 in vessels from hypertensive rats. Spironolactone reduced the number of neutrophils; however, in co-presence with high physical activity this effect was blunted. In conclusion, although high physical activity has beneficial effects in normotensive rats, this does not predict similar beneficial effects in the concomitant presence of hypertension and care has to be taken on interactions between pharmacological approaches and high physical activity in hypertensives.

## Introduction

A high level of physical activity is recommended to reduce the risk of hypertension and related diseases (1). This assumption is based at least in part on findings showing that hypertension is associated with sterile vascular inflammation and mobilization of pro-inflammatory leukocytes (2, 3). Exercise may attenuate some of these vascular stress factors (4). In most cases, hypertension is characterized as primary hypertension that means no organic causes can be found that are responsible for the development of high blood pressure. Nearly 90% of all hypertensive patients belong into this category. Among them, in ~60% genetic factors are the triggers and underlying causes of hypertension (5). Spontaneously hypertensive rats (SHRs) are a suitable animal model that mimics most of these characteristics (6, 7). The animals develop arterial hypertension during adulthood, behave normal, and healthy for a long time and finally develop end-organ damages (i.e., heart, kidney) at an advanced age. According to the current knowledge, the development of high blood pressure levels in SHRs is based on polygenic causes.

Exercise is considered as healthy and protective, however, the extent to which physical activity can be considered reliable preventive and therapeutic options for cardiovascular patients still cannot be answered with any certainty (8). Exercise activates the sympathetic nervous system (SNS), the angiotensin-aldosterone system (RAAS), and induces pro-inflammatory effects characterized by increased release of interleukin (IL)-6 and mobilization of leukocytes in rodents and humans (9–14). The induction of the SNS and RAAS is required to adapt the circulation to the increased energy demand during the time of exercise, whereas the induction of pro-inflammatory processes are required for post-exercise repair processes of skeletal muscles and the vasculature. Specific interest has been given to the release of plasminogen activator inhibitor (PAI)-1 during exercise (9, 15). The vascular expression of PAI-1 has been directly linked to the RAAS as it depends on angiotensin II via activation of angiotensin type 1 (AT1) receptors (16). Of note, plasma concentrations of angiotensin II raise in SHRs compared to normotensive rats during night and are responsible for PAI-1 expression (17). Consequently, PAI-1 plasma levels are higher in hypertensives (18). Furthermore, it could already be shown that aldosterone damages blood vessels via PAI-1 (19).

In the post-exercise phase, however, levels of sympathetic activation decrease as indicated by lower resting heart rate, as well as aldosterone levels (20); on the other hand anti-inflammatory and anti-oxidative effects occur to some extent (4, 21, 22). Therefore, exercise has the ability to further stress the vasculature during exercise and to protect it against the consequences of the persistent high blood pressure in resting periods. In addition to PAI-1, changes in the transcriptome of the vasculature are related to inflammation, nitric oxide metabolism, oxidative stress, extracellular matrix, and metabolism (23–25). These effects are at least in part mediated by the interaction of blood cells with vascular cells and thus includes adherence of blood cells to the vascular wall, migration of cells into the vascular wall, and release of cytokines from such cells. Exercise may further modify the biological activity of such cells.

In the current study we first characterized the impact of voluntary running wheel activity on circulating lymphocytes counts and the subsequent modification of molecular adaptation of the vascular wall in normotensive rats to define the effects of running wheel activity on these parameters. Next, we compared these effects to those obtained in SHRs to investigate whether potentially beneficial effects in normotensive rats remain active in SHRs. Finally, we compared the effects of running wheel activity in SHRs to those of aldosterone blockade and hypothesized that both treatments (high physical activity and aldosterone blockade) can be regarded as safe preventive and therapeutic modalities in the concomitant presence of hypertension. The aim of the study was to investigate whether co-morbidities like essential hypertension affect the response of rats to high physical activity. As inflammatory processes of the vascular wall are critical for the further progression of hypertensive desease we is inflammatory processes of the vascular wall are critical for the further progression of hypertensive desease we focused on mobilization of white blood cells and the impact on molecular adaptation in the vascular wall.

## Materials and Methods

The investigations are in agreement with the “Guide for the Care and Use of Laboratory Animals” purchased by the US National Institute of Health (NIH Publication No. 85-23, revised 1996). The study was approved by the local authorities (RP Gießen; V 54–19 c 20 15 h 01 GI 20/1 Nr. 76/2014 and GI 20/1 Nr.77/2014).

### Animal Model

At the start of their 6th week of life female Wistar rats (Hsd:WI) or SHR (NHsd) were randomized selected and kept either under standard conventional housing conditions or received free access to a running wheel during the night for a period of 10 months. In two groups (sedentary and running) SHRs received spironolactone for the last 4 months (50 mg/kg/day via the feed), in another group the running wheels were removed over the last 4 months. Running distance, running time, kidney function and blood pressure values were reported earlier for most of the rats; (26, 27).

### Study Design

**Figure 1:**
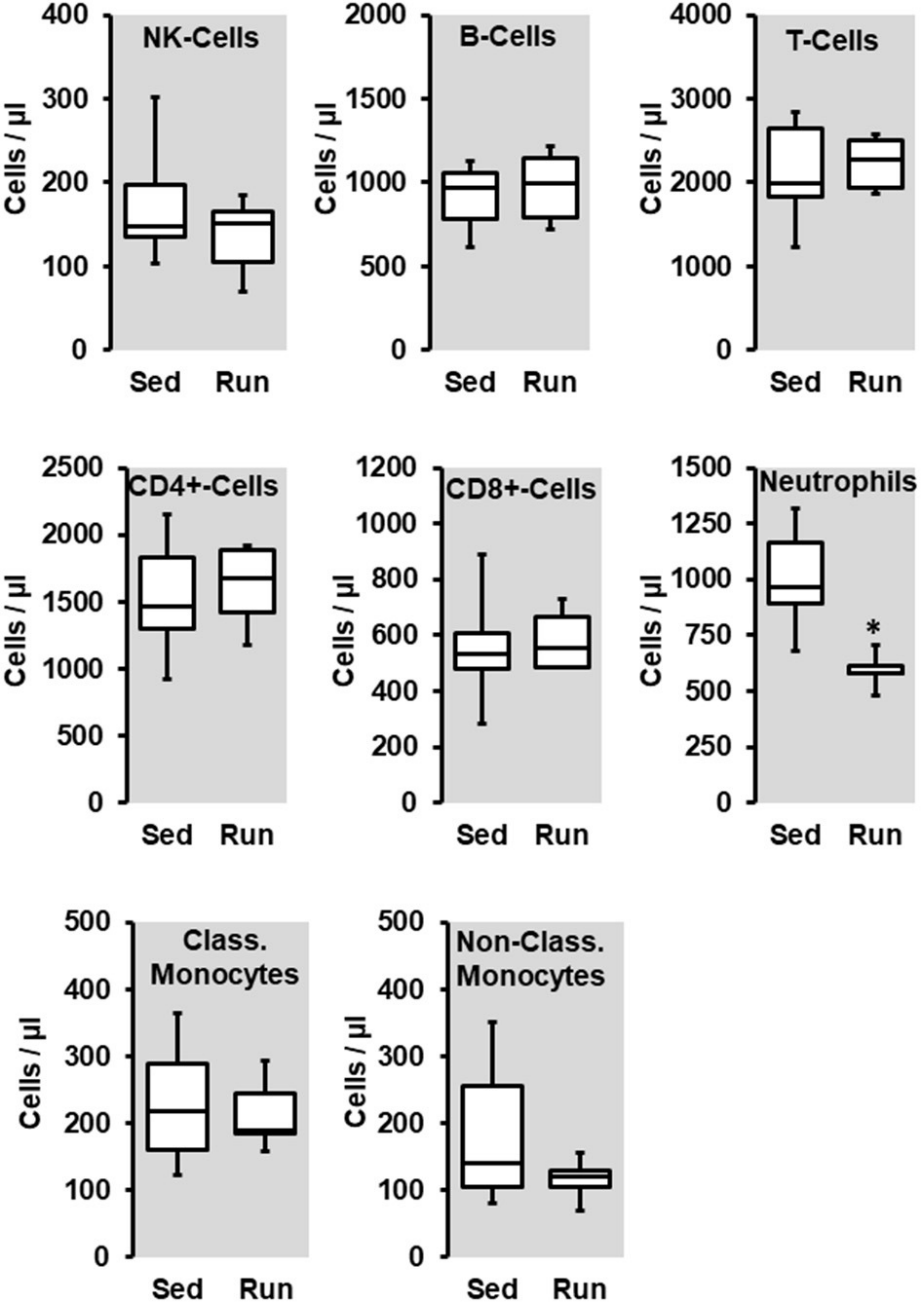
Effect of 10 months voluntary running wheel activity (Run) on blood cell concentration in normotensive rats compared to sedentary controls (Sed). Box plots represent 25, 50, and 75 percentiles with 5 and 95 percentiles given as whiskers. *p < 0.05; unpaired *T*-Test.

**Figure 2:**
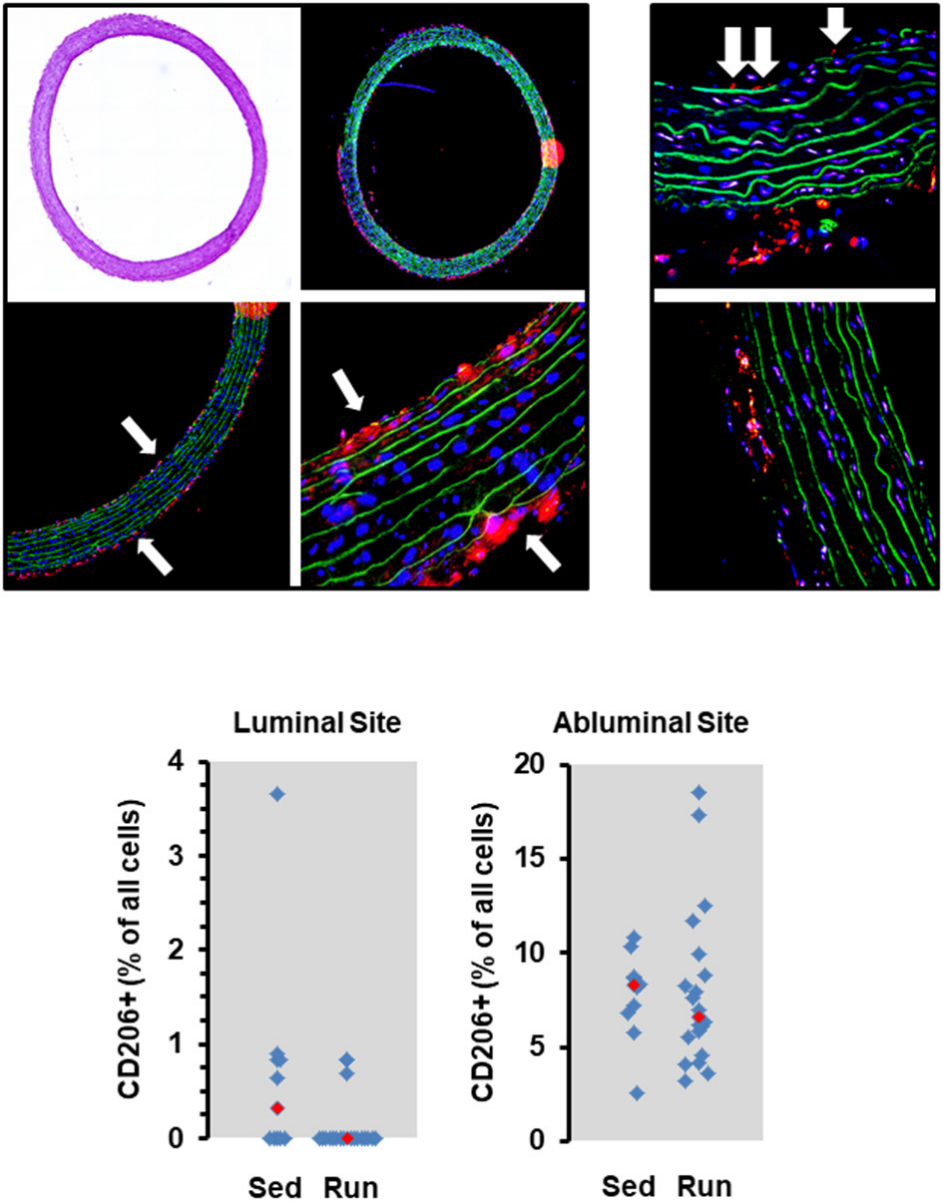
Histological cross sections from aortae of normotensive rats and the effect of 10 months voluntary running wheel activity. Top left: Overview of an aorta by light microscopy, confocal laser microscopy, and two different magnifications used to count the number of CD206^+^ cells (in red) normalized to the total cell number as calculated by DAPI-stained nuclei in blue, and lamina elastic (in green). Top right: Examples of intraluminal located CD206^+^ cells (white arrows) from sedentary rats and from rats after 10 months running wheel activity. Bottom: Quantification of different slices from *n* = 5 rats each group. Red rectangles indicate the median, blue rectangles individual data points.

**Figure 3:**
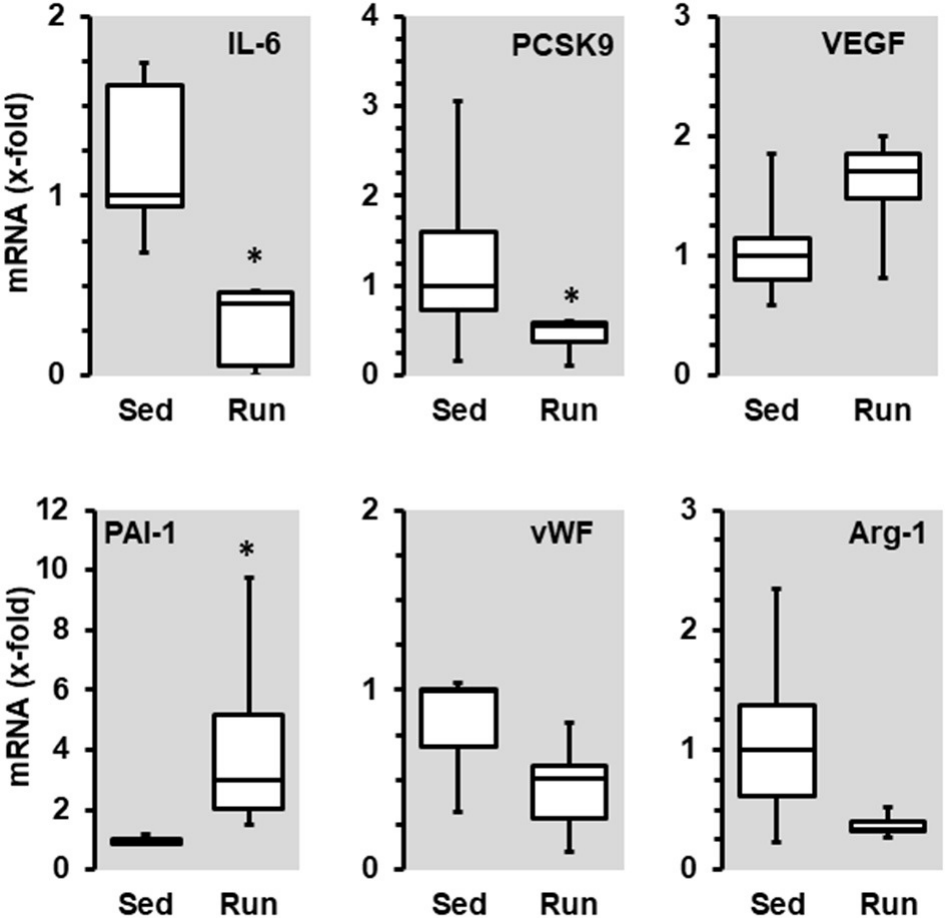
Effect of 10 months voluntary running wheel activity (Run) on mRNA expression of selected genes in normotensive rats compared to sedentary controls (Sed). Selection criteria are differences in median expression of more than 50% or *p*-values below 0.05. Box plots represent 25, 50, and 75 percentiles with 5 and 95 percentiles given as whiskers. *p < 0.05; unpaired *T*-Test.

This study consists of three experimental parts that were performed in line:

First part: Wistar Sedentary (*n* = 6) and Wistar Running (*n* = 6). These data are given in Figures 1–**3**.

**Figure 4:**
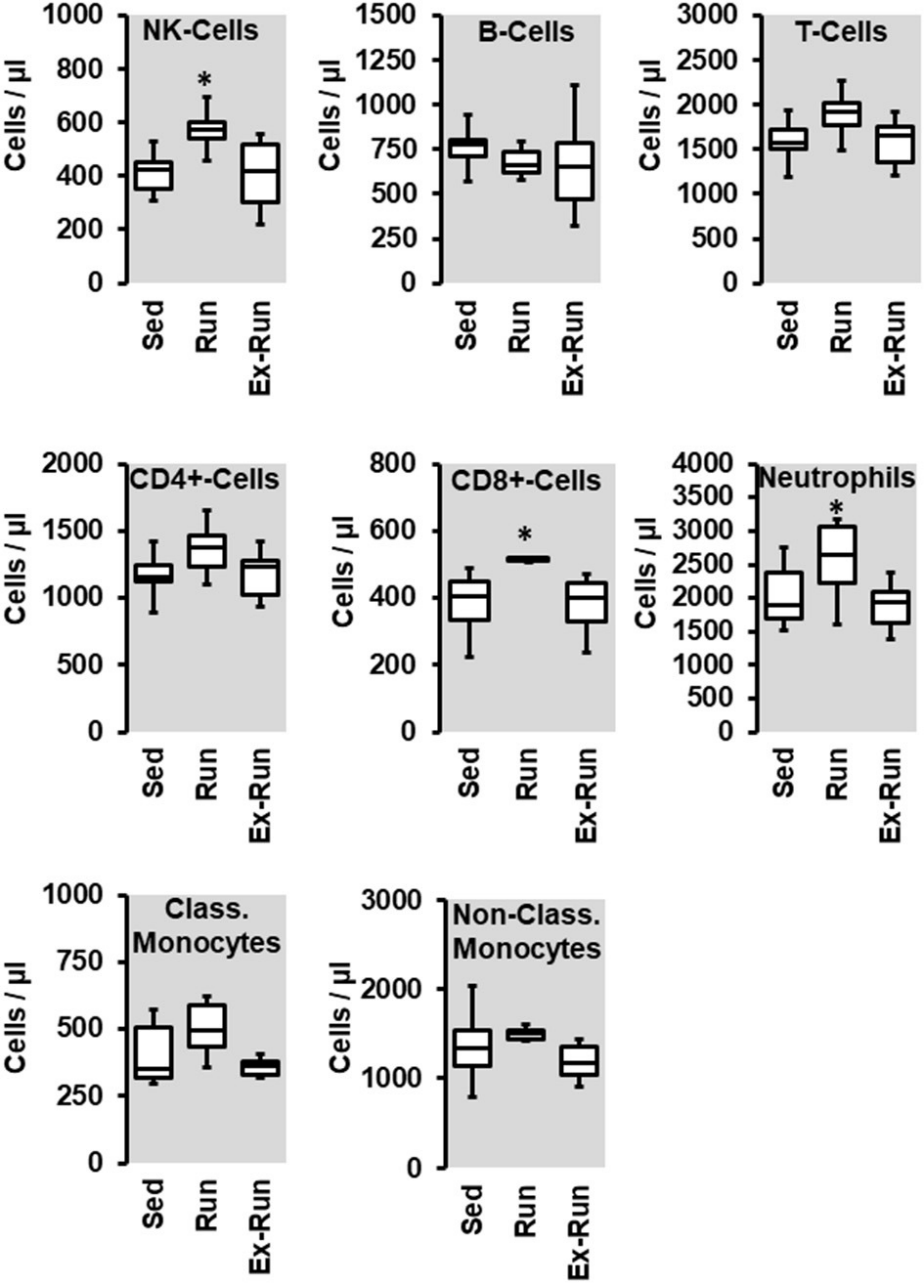
Effect of 10 months voluntary running wheel activity (Run) on blood cell concentration in hypertensive rats compared to sedentary controls (Sed) and SHRs running for 6 months with subsequent 4 months cessation (Ex-Run). Box plots represent 25, 50, and 75 percentiles with 5 and 95 percentiles given as whiskers. *p < 0.05; One-Way ANOVA with subsequent Student-Newman-Keuls *post-hoc* analysis.

**Figure 5:**
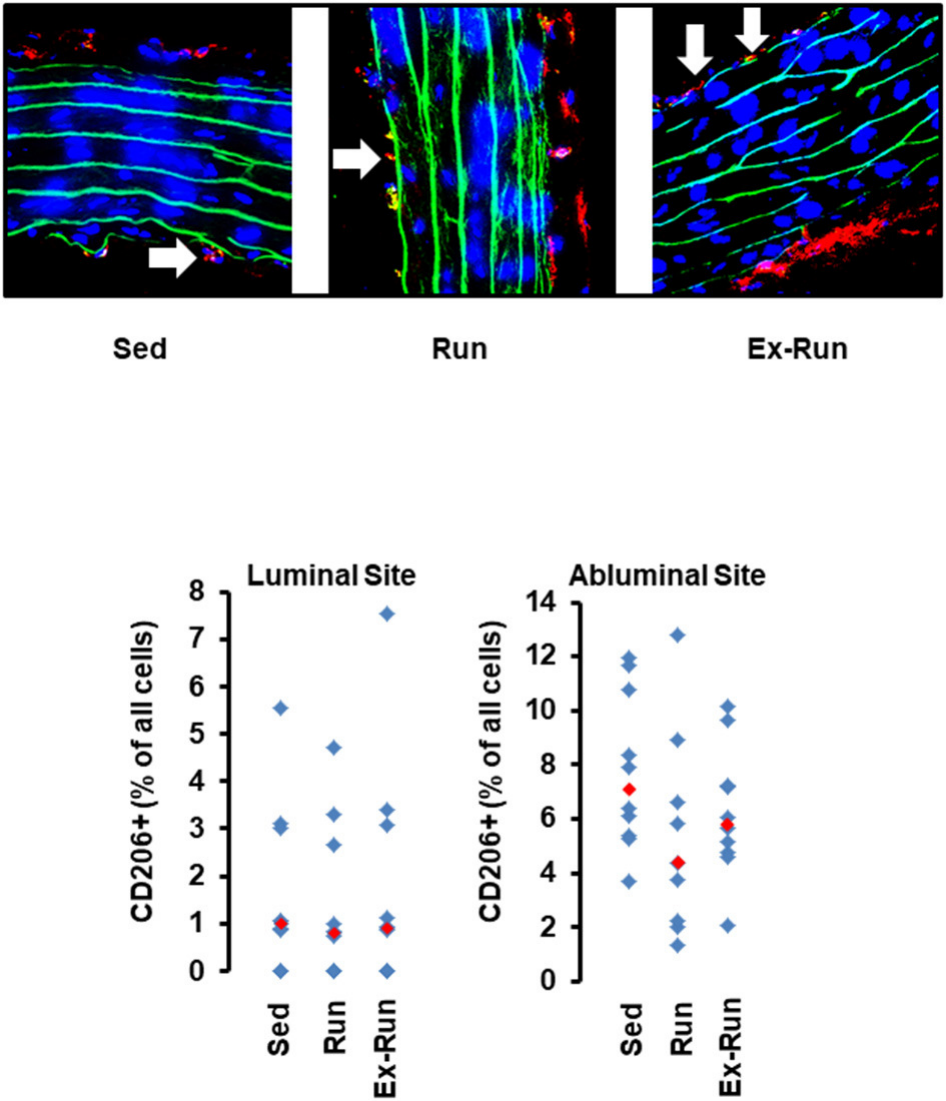
Histological cross sections from aortae of hypertensive rats and the effect of voluntary running wheel activity. Top: Representative slights from confocal laser microscopy used to count the number of CD206^+^ cells (in red) normalized to the total cell number as calculated by DAPI-stained nuclei in blue. Lamina elastica is seen in green. Bottom: Quantification of different slices from *n* = 5 rats each group. Red rectangles indicate the median, blue rectangles individual data points.

**Figure 6:**
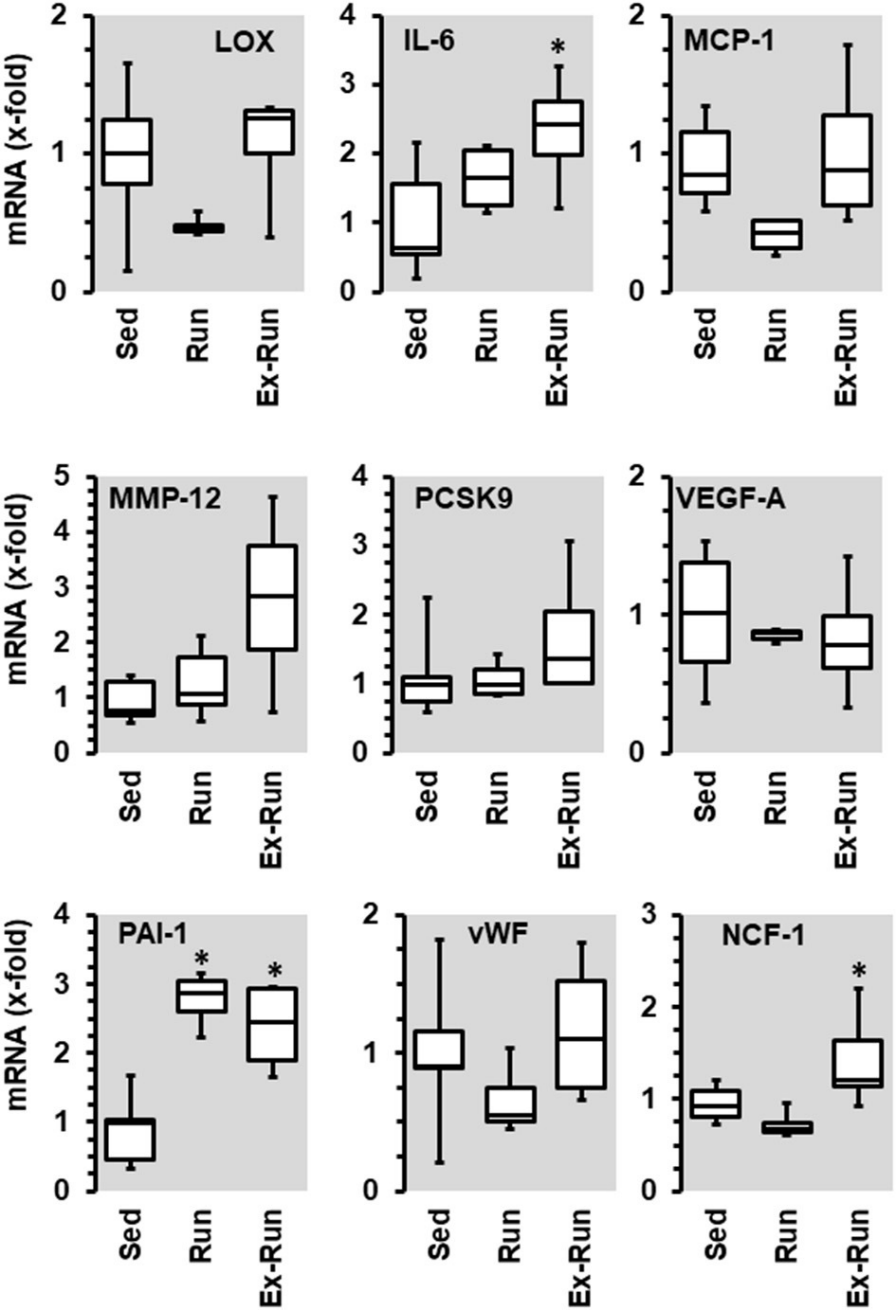
Effect of 10 months voluntary running wheel activity (Run) on mRNA expression of selected proteins in hypertensive rats compared to sedentary controls (Sed) and SHRs running for 6 months with subsequent 4 rmonths cessation (Ex-Run). Box plots represent 25, 50, and 75 percentiles with 5 and 95 percentiles given as whiskers. *p < 0.05; One-Way ANOVA with subsequent Student-Newman-Keuls *post-hoc* analysis.

Second part: SHR sedentary (*n* = 5), SHR running (*n* = 6). SHR ex running (*n* = 6). These data are given in **Figures 4**–**6**.

**Figure 7:**
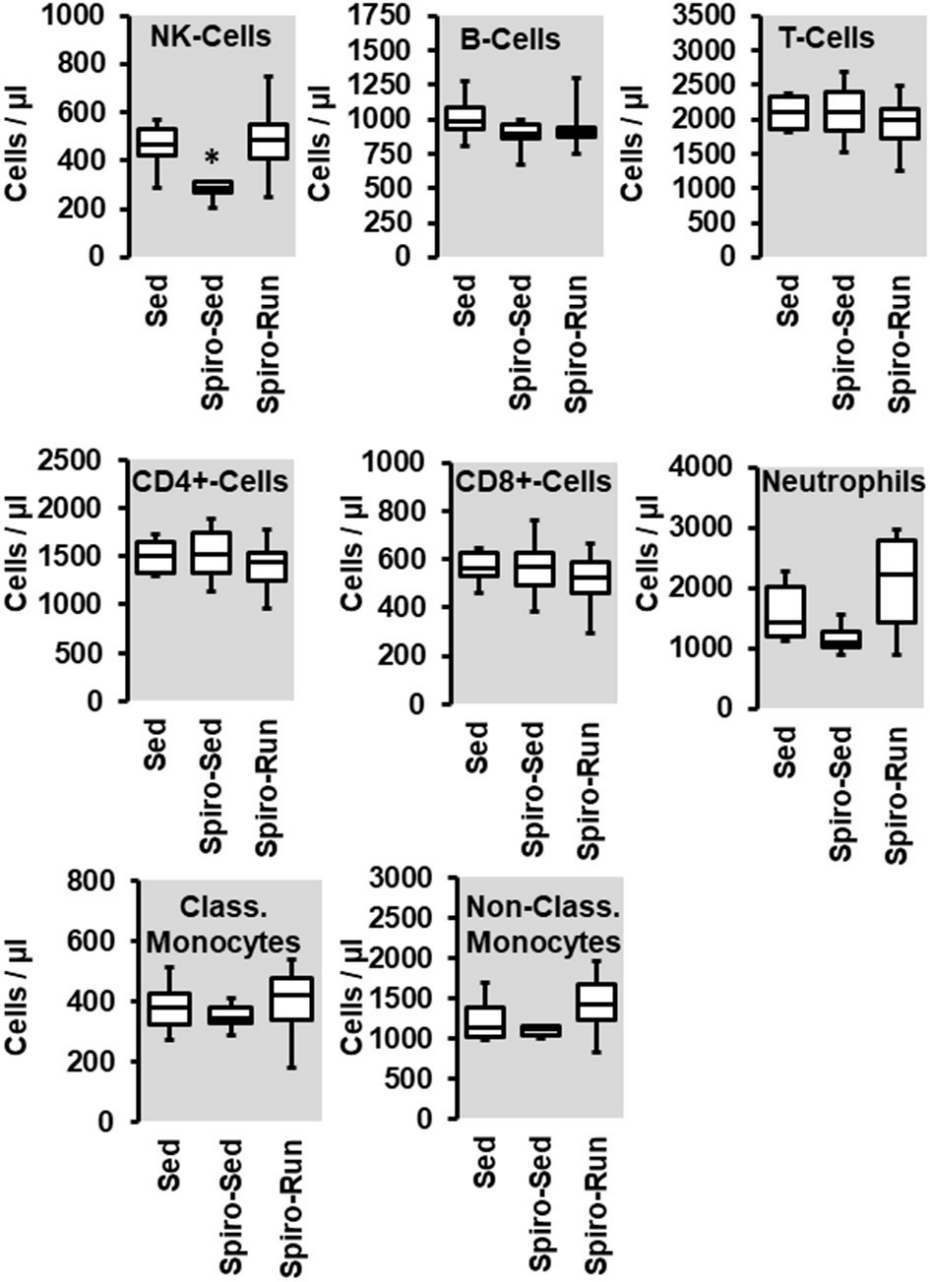
Effect of spironolactone (Spiro) given for the last 4 months and of voluntary running wheel activity (Run) on blood cell concentration in hypertensive rats compared to sedentary controls (Sed). Box plots represent 25, 50, and 75 percentiles with 5 and 95 percentiles given as whiskers. *p < 0.05; One-Way ANOVA with subsequent Student-Newman-Keuls *post-hoc* analysis.

**Figure 8:**
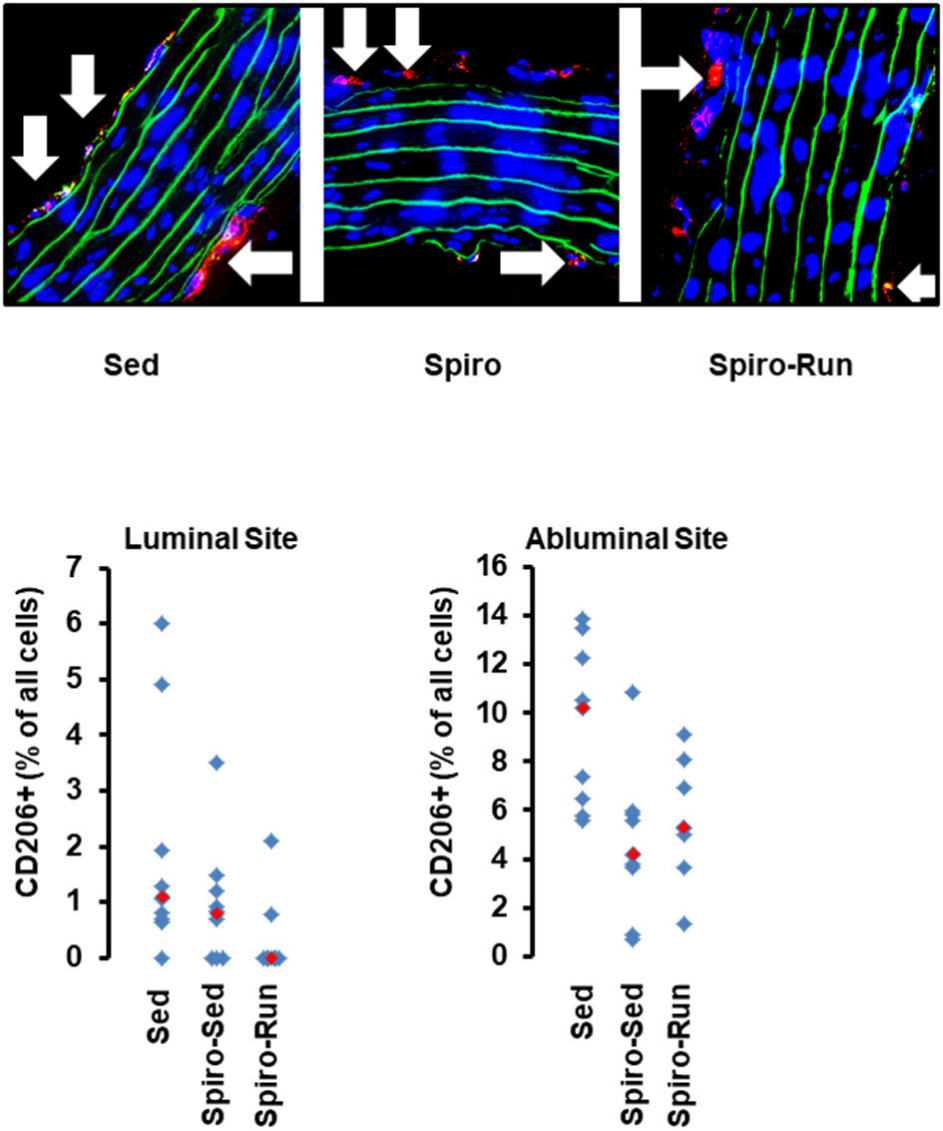
Histological cross sections from aortae of hypertensive rats treated with spironolactone (see Figure 7). Top: Representative slights from confocal laser microscopy used to count the number of CD206^+^ cells (in red) normalized to the total cell number as calculated by DAPI-stained nuclei in blue. Lamina elastica is seen in green. Bottom: Quantification of different slices from *n* = 5 rats each group. Red rectangles indicate the median, blue rectangles individual data points.

**Figure 9:**
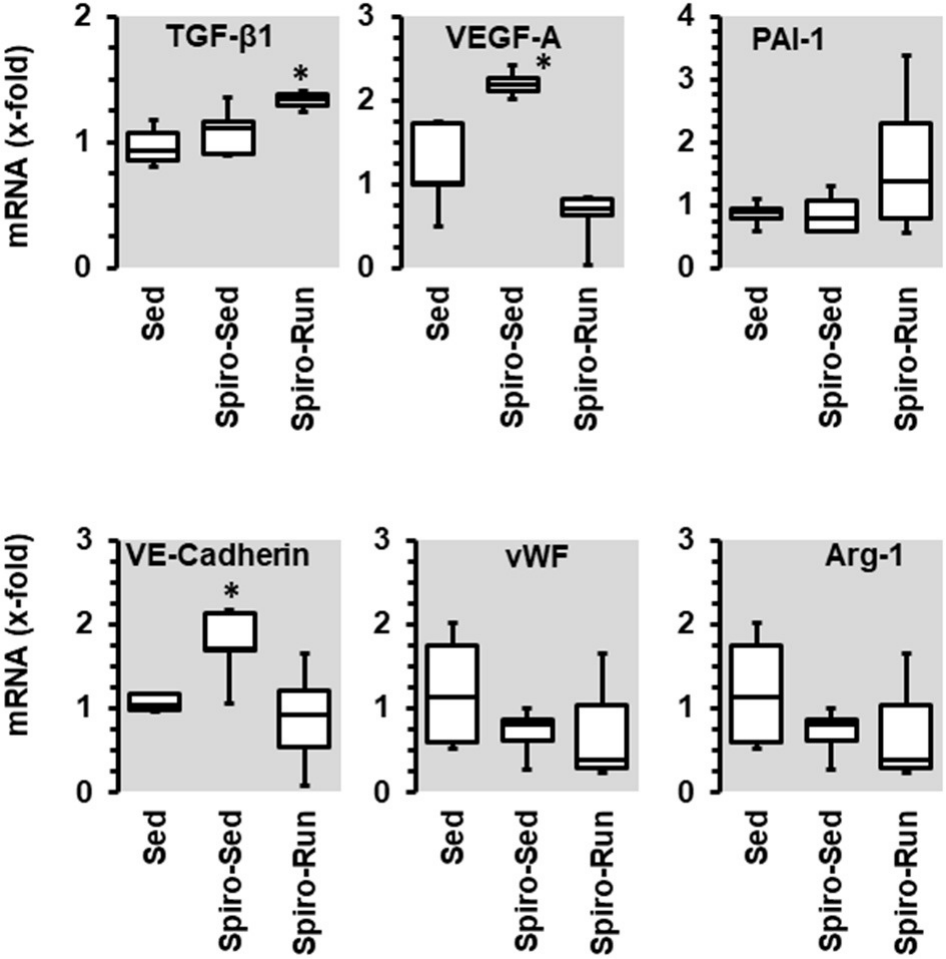
Effect of 10 months voluntary running wheel activity (Run) and spironolactone (Spiro) on mRNA expression of selected proteins in hypertensive rats compared to sedentary controls (Sed). Box plots represent 25, 50, and 75 percentiles with 5 and 95 percentiles given as whiskers. *p < 0.05; One-Way ANOVA with subsequent Student-Newman-Keuls *post-hoc* analysis.

Third part: SHR sedentary (*n* = 5), SHR Spiro-Sedentary (*n* = 6), SHR Spiro-Run (*n* = 6). These data are given in **Figures 7**–**9**.

In the second and third part, one animal from the sedentary group were not considered for further analysis, due to excessive right ventricular hypertrophy.

### Blood Sampling and FACS Analysis

Blood samples were collected 1 week before rats were sacrificed by punctuation of the tail vein. Approximately 500 μl of peripheral blood were collected in BD Microtainer Plastic Capillary Blood Cellectors with Dipotassium EDTA. Erythrocytes were lysed with 1 ml of RBC Lysis buffer solution (eBioscience, Cat.-No. 00-4333-57) for 5 min at 37°C. The lysis reaction was neutralized with 10 ml of saline. Peripheral blood leukocytes were collected by centrifugation at 500 g for 5 min. The resulting pellets were resuspended in 400 μl of cold flow cytometry staining buffer solution and processes for flow cytometry. Samples were incubated for 30 min on ice with TruStain fcX to block Fc receptors and subsequently incubated with a cocktail of fluorophore-labeled monoclonal antibodies for 1 h at 4°C protected from light. Following a wash with ice-cold saline, samples were finally resuspended in 400 μl of cold Flow Cytometry Staining Buffer Solution. Different antibodies were used to define the cell populations as indicated in Table 1. Data were acquired on an LSRII flow cytometer (BD Bioscience) and analyzed with FlowJo software, version 7.6.3.

**Table 1:** Number of circulating blood cells per μl in 10 months old female rats.

	**Wistar**	**SHR**	**p-Value**
NK-cells (CD161^+^)	174 ± 65	404 ± 66	0.000018
B-cells (CD45R^+^)	914 ± 186	748 ± 100	0.058594
T-cells (CD3^+^)	2,112 ± 570	1,587 ± 205	0.096548
T-cells (CD3^+^/CD4^+^)	1,533 ± 406	1,176 ± 145	0.108968
T-cells (CD3^+^/CD4^+^/CD8a^+^)	14 ± 5	9 ± 3	0.011261
T-cells (CD3^+^/CD4^+^/CD8a^−^)	9 ± 5	10 ± 2	0.587641
T-cells (CD3^+^/CD8a^+^)	555 ± 184	372 ± 80	0.022427
Neutrophils (CD43^+^)	1,006 ± 221	1,985 ± 405	0.000416
cMonocytes (CD11b^+^/CD172a^+^/CD43low)	229 ± 85	371 ± 80	0.007371
ncMonocytes (CD11b^+^/CD172a^+^/CD43high)	183 ± 102	1,224 ± 253	<0.000000

*NK, natural killer; c, classical; nc, non-classical; n = 6 Wistar and n = 10 SHR. p-values are calculated by unpaired T-Tests*.

### Histology

To visualize leukocyte (CD206^+^ cells; M2 macrophages) adhesion to the endothelium or transmigration through the vessel wall sectioned tissue samples of the aortae were analyzed directly with laser scanning confocal microscopy. The focus on M2 macrophages was chosen because the greates difference between circulating white blood cells between normotensive and hypertensive rats was seen for non-classicakl monocytes. Aortae were removed and frozen in Tissue-Tek O.C.T. compound. Samples were then sectioned axially into 5-μm slices, fixed in 4% paraformaldehyde for 5 min, and washed 3 times in saline for 3 min. All sections were incubated with the primary antibody diluted in blocking solution overnight at 4°C. Sections were washed and reincubated with anti-Rat Cy3 secondary antibody for 1 h at room temperature followed by washing. Nuclei were stained with a dilution of 4′6′-diamidino-2-phenylindole, dihydrochloride, DAPI. Cover slips were mounted using a drop of Mowiol 4–88 mounting medium.

### Real Time RT-PCR

Total RNA was isolated from aortic tissues using peqGOLD TriFast according to the manufacturer's protocol. To remove genomic DNA contamination, isolated RNA samples were treated with 1 U DNase/μg RNA for 15 min at 37°C. One microgram of RNA was used in a 10 μl reaction to synthesize cDNA using Superscript RNase H Reverse Transcriptase and oligo(dT) as primers. The CFX Connect Real Time PCR Detection System, supplied by Bio-Rad, was used to perform quantitative PCRs as described before (27). The sequences of the primers used in this study are indicated in Table 2. Quantification (ΔΔ*C*_T_ method) was analyzed as described before (28).

**Table 2:** Primers used for this study.

**Gene**	**Forward**	**Reverse**
*ARG1*	GGA AGC ATC TCT GGC CAC GCC	CAC CGG TTG CCC GTG CAG AT
*B2M*	GCC GTC GTG CTT GCC ATT C	CTG AGG TGG GTG GAA CTG AGA C
*IL6*	TGG TCA AAG ACG TTC ACG GT	CTC TGC TAG CCA AGG AGT GC
*LOX*	ACA ACC GCA CTG CCT CTG CC	GCC TTG AGG CTC CAT CGC CG
*CCL2*	TCA CGC TTC TGG GCC TGT TGT	GCC TTG AGG CTC CAT CGC CG
*MMP12*	GC AGC TGT CTT TGA TCC AC	GCA TCA ATT TTT GGC CTG AT
*NCF1*	GCA CCA CCT CGC AGG TCG AC	GCG CTG CTG CAG GAA TCG GA
*SERPINE1*	CCT CCT CAT CCT GCC TAA G	TGC CGC TCT CGT TCA C
*PCSK9*	TTG AAC AAA CTG CCC ATC GC	CCC AAC AGG TCA CTG CTC AT
*TGF-β_1_*	ATT CCT GGC GTT ACC TTG G	CCT GTA TTC CGT CTC CTT GG
*VEGF-A*	TGC CCC TAA TGC GGT GTG CG	GGC TCA CAG TGA ACG CTC CAG G
*CDH5*	CCA GAA TTT GCC CAG CCC TA	GTC CTC GTT CTT CAG GGC AA
*vWF*	AAG ATG GCA AGA GAG TGG GC	CCG TAG GCC TCA CTG GAA AG

*The genes are encoding for the following proteins: ARG1, arginase-1; B2M, β2-Microglobulin; IL6, Interleukin-6; LOX, lysyl oxidase; CCL2, Monocyte Chemotactic Protein 1; MMP12, Matrix Metalloproteinase-12; NCF1, Neutrophil cytosol factor 1; SERPINE1, Plasminogen-activator-inhibitor; PCSK9, Proprotein Convertase Subtilisin/kexin type 9; TGF β_1_, Transforming growth factor β_1_; VEGF-A, Vascular Endothelial Growth Factor A; CDH5, VE-Cadherin; vWF, von Willebrand Factor*.

### Statistics

Data on cell numbers and mRNA expression are presented as box plots with whiskers, where the 25, 50, and 75 percentiles are given by boxes and that of the 5 and 95 percentiles are given by whiskers. *P*-values below 0.05 are indicated by ^*^. *P*-Values were calculated by unpaired *T*-Tests (Figures 1, **3**, Table 1) or ANOVA with Student-Newman-Keuls *post-hoc* analysis (**Figures 4**, **6**, **7**, **9**). All data points and the median (in red) are given for histological quantification (Figures 2, **5**, **8**).

## Results

### Effect of Voluntary Running Wheel Exercise on the Number of Circulating Lymphocytes, the Adherence of Lymphocytes to the Vascular Wall, and Transcriptional Adaptation of the Vasculature in Normotensive Rats

The number of circulating natural killer cells (NK-cells), B cells, CD4^+^ and CD8^+^ T cells, neutrophils, and monocytes was analyzed in blood samples from normotensive rats shortly before the end of the trial. Ten months running wheel exercise had no effect on these counts with neutrophils being an exception. The amount of circulating neutrophils was ~40% lower in rats with running wheel activity compared to their sedentary counterparts (Figure 1). Although the amount of circulating monocytes did not vary between both groups the number of cells that migrate into the vessel wall as well as the number of cells that adhere to the vascular endothelium may differ. Therefore, the number of CD206^+^ cells was quantified in histological slices. However, there was again no difference at all (Figure 2).

In contrast to these moderate effects, running wheel activity influenced transcriptional expression in the vascular tissue. The mRNA expression of interleukin (IL)-6, proprotein convertase subtilisin/kexin type 9 (PCSK9), von Willebrand factor (vWF), and arginase-1 was reduced in vessels from animals with access to free-running wheels (*p* < 0.05 for IL-6 and PCSK9), in contrast, the expression of vascular endothelial growth factor (VEGF)-A and PAI-1 (*p* < 0.05) was induced (Figure 3). Overall, the down-regulation of IL-6 and PCSK9 may contribute to a reduced risk of inflammatory processes in the overall vasculature, whereas the reduction of vWF may reduce the risk of thrombosis and the reduction of arginase-1 may favor NO formation, while induction of VEGF-A may favor proliferation of endothelial cells as required for repair processes and PAI-1 may contribute to vascular wall stabilization. Overall, the observed transcriptional changes promote a healthy phenotype of the vasculature.

### Effect of Voluntary Running Wheel Activity on the Number of Circulating Lymphocytes, the Adherence of Lymphocytes to the Vascular Wall, and Transcriptional Adaptation of the Vasculature in Spontaneously Hypertensive Rats

Next we compared the number of circulating lymphocytes between sedentary normotensive rats and SHRs (Table 1). Compared to normotensive rats, SHRs have increased number of circulating leukocytes, mainly NK-cells (2.32-fold), neutrophils (1.97-fold), and non-classical monocytes (6.69-fold). In normotensive rats, running activity was sufficient to reduce the number of neutrophils. Therefore, running wheel activity should also normalize the high number of neutrophils in SHRs. However, in SHRs running wheel activity caused an even further increase in the number of circulating neutrophils as well as a strong rise in the number of NK-cells and CD8^+^ T cells, that was not observed in normotensive rats (Figure 4). The effect of running wheel activity was indeed responsible for these effects as cessation of running activity during the last 4 months completely normalized these increases (Figure 4). No differences between the number of CD206^+^ cells in the aorta wall or adherence to the aortic endothelium was observed between groups of SHRs (Figure 5).

Voluntary running wheel activity reduced the mRNA expression of lysyl oxidase (LOX) in the aorta tissue of SHRs as well as that of monocyte chemotactic protein (MCP1), vWF, and Ncf-1, but increased that of PAI-1 (Figure 6). Among these changes, only the reduction of vWF and the induction of PAI-1 were similar to that observed in normotensive rats. Importantly, the effects of running wheel activity on mRNA expression of LOX, MCP1, and vWF but not of PAI-1 mRNA expression were all reversible as shown by the cessation of running activity. Of note, termination of the training program increased the expression of IL-6, matrix metalloproteinase (MMP)-12 and neutrophil cytosol factor (NCF)-1 (=p47 phox). In contrast to normotensive rats, running activity had no effect on the mRNA expression of VEGF-A and PCSK9 in SHRs.

### Effect of Aldosterone Inhibition on the Number of Circulating Lymphocytes, the Adherence of Lymphocytes to the Vascular Wall, and Transcriptional Adaptation of the Vasculature in Spontaneously Hypertensive Rats and Its Interference With Running Wheel Activity

As already shown, SHRs have higher levels of NK-cells, neutrophils, and monocytes. Among them, aldosterone blockade by spironolactone was sufficient to reduce the number of NK-cells and at least in part also that of neutrophils but did not modify the number of monocytes (Figure 7). However, combined with voluntary running wheel exercise, spironolactone was ineffective to reduce the amount of NK-cells and neutrophils (Figure 7). Spironolactone treatment strongly reduced the number of CD206^+^ cells that was found in the aortae of these rats (Figure 8). The combination of high physical activity and spironolactone reduced specifically the number of CD206+ cells at the luminal side.

Spironolactone treatment increased the expression of VEGF-A and VE-cadherin, an effect that was not seen when the administration of spironolactone was combined with running activity. Spironolactone treatment reduced the expression of arginase-1 and vWF but did not modify PAI-1. In the combination of spironolactone and running wheel activity the anti-inflammatory and pro-fibrotic cytokine TGF-β_1_ was induced (Figure 9).

## Discussion

**Figure 10:**
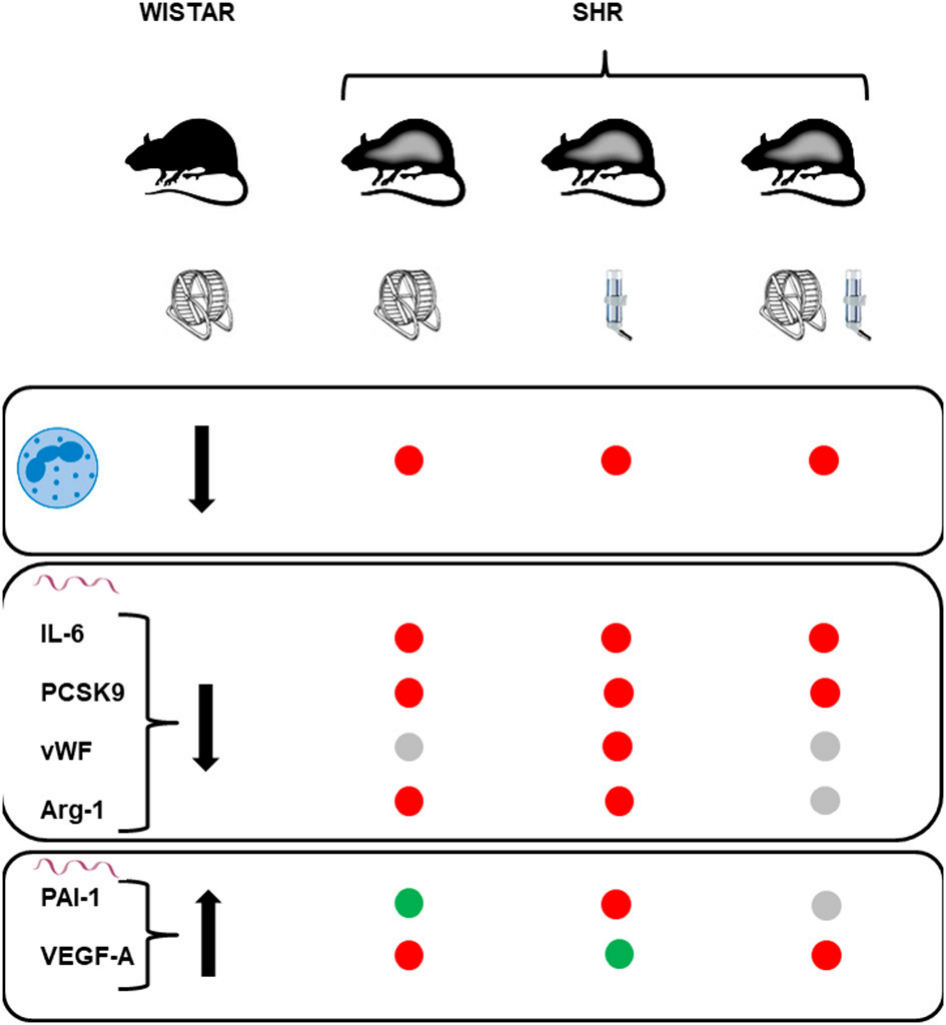
Summary and comparison of the effects of life-long high physical activity in normotensive rats (Wistar) to those seen on these parameters in sponatenously hypertensive rats (SHR). High physical activity decreases the number of circulatin neutrophils (first raw), decreases the mRNA expression of IL-6, PCSK9, vWF, and arginase-1 (second raw), and increases the mRNA expression of PAI-1 and VEGF-A (third raw). Red points in the SHR columns stand for lack of similar effect, gray points for similar effects but of minor strength (*p* > 0.05), and green points indicate similar responses.

Our study was aimed to investigate whether protective effects of high physical activity seen in normotensives can be translated into similar effects in the presence of essential hypertension, as a co-morbidity. In normotensiove rats we found a couple of strong effects of exercise on white blood cell number (decrease in neutrophile cell counts), low monocyte adherence at the vascular wall, and in the vascular wall reduced expression of IL-6, PCSK9, vWF, and arginase 1 and increased expression of PAI-1 and VEGF-A. In most cases, these adaptations to high physical activity can be considered as protective and similar adaptation in hypertensive rats should be considered as important for the progression of the disease. This was however not the case. In hypertensive rats we did not only found little evidence for similar adaptations but additive stress by high physical activity. In contrast to high physical activity, pharmacological treatment with spironolactobe was sufficient to reverse some of the mal-adaptaive effects in SHRs that were analyzed here. Examples are give by NK-cells counts, cell adheasion, and VEGF-A. However, with the exception of cell adhesion this was lost when spironolactobne was combined with high physical activity. Therefore, this study suggests that hypertension-dependend mechanisms overrun the protective effects of high physical activity in rats and that these mechanisms in SHRs are aldosterone independent. Figure 10 compares the effects of high physical activity found in normotensive rats to those found in SHRs with either high physical activity, spironolactobe, or a combination of both.

A participation of inflammatory processes affecting the vasculature in the context of hypertension is well-established and inflammatory blood cells contribute to this process. In this study we confirmed this hypothesis by comparison of the number of circulating white blood cells between normotensive and spontaneously hypertensive rats. The numbers of several leukocytes that are related to inflammation were significantly increased in SHRs. Among them, we found increased counts for NK-cells, neutrophils, and monocytes, mainly non-classical monocytes, but also a reduced counts for CD8^+^ of T cells. Constitutive high physical activity is expected to exert anti-inflammatory effects. We found indeed that high physical activity decreased the number of circulating neutrophils in normotensive rats. Acute bursts of exercise normally increase the mobilization of neutrophils although decreasing their activity. However, in the long-term adaptation to high physical activity normotensive female rats had lower neutrophil counts than sedentary female rats. Similarily, in several long-term studies reduced neutrophil counts were described due to exercise in overweight women as well as in untrained men (29–32). However, in SHRs a similar effect of high physical activity was absent. On the very contrary, the number of neutrophils and in addition that of NK cells further increased whereas the number of CD8^+^ T cells was normalized. All these effects are directly dependent on high physical activity as they were reversed when the running wheel was removed after 6 months of training. It can therefore be concluded that the effect of high physical activity in normotensive rats does not predict the outcome in SHRs. Moreover, as white blood cells are directly associated with blood pressure, suggesting a causative relationship, high physical activity does not protect against blood cell-vasculature interaction (3).

Based on the PCR analysis performed in this study on aortic tissue, running wheel activity triggers several favorable effects in the vasculature such as reduction of the expression of the pro-inflammatory cytokine IL-6, of the pro-thrombotic vWF, and of the NOS competitor arginase-1. Similarly, higher physical activity is associated with lower plasma levels of IL-6, vWF, and arginase 1 in men (33–35). Furthermore, running wheel activity reduced the vascular expression of PCSK9 often associated with oxidative stress (36). In contrast, the vascular expression of PAI-1 and VEGF-A was increased. The effect of exercise on PAI-1 has repeatedly been analyzed leading to divergent results. Exercise decreased PAI-1 expression (18, 37), does not affect PAI-1 expression (38), or increases PAI-1 expression (9, 15). Vascular effects of PAI-1 depend on the activation of components of the RAAS (16, 17, 19). Consistent with these findings, running-induced PAI-1 expression was lowest in the co-presence of spironolactone in our study. An exercise-dependent upregulation of VEGF-A is consistent with many other studies and has been conformed in various tissues (39). In SHRs the effects on the transcriptional adaptation were much more complex compared to normotensive rats. Similar to normotensive rats, vWF was down-regulated and PAI-1 was induced in SHRs performing high physical activity. Moreover, in the SHRs, aortic expression of LOX-1, and MCP-1 was reduced. While the expression of LOX-1 is required for maturation of collagen fibers and thus stabilization of the vascular wall, MCP-1 is a pro-inflammatory cytokine. Interestingly, most of the changes were reversed when the running activity was ceased, except that of PAI-1. In this context, MCP-1 is required for accumulation and migration of monocytes/macrophages and we found in our SHRs indeed an inverse relationship between MCP-1 expression and macrophages at the abluminal side of the aorta (40).

In SHRs we also analyzed whether the running-induced adaptations are reversible. Cessation of high physical activity after 6 months indicated a direct correlation between activity and alterations in NK cells, CD8^+^ cells, and neutrophils in SHRs. A similar trend was found for the number of CD206^+^ cells at the abluminal side of the aorta. On the mRNA level, downregulation of LOX-1, MCP-1, vWF, and NCF-1 were also reversible. In contrast, the activity-dependent expression of PAI-1 was not reversed. Furthermore, cessation worsens the expression of IL6 and NCF-1. This may indicate that these adaptations are linked to high physical activity in the early adulthood and adapations to this life-style rather than direct effects of high physical activity.

In contrast to running wheel activity the number of circulating NK-cells and at least in part that of neutrophils was reduced in SHRs receiving spironolactone suggesting that mobilization of NK-cells is at least in part aldosterone-dependent. However, in spironolactone treated SHRs performing running wheel activity these changes were absent. The data suggest that the exercise-dependent effect on NK-cells is aldosterone independent. Furthermore, spironolactone increased the expression of VEGF-A and VE-cadherin but again not in combination with running wheel activity. This suggests that aldosterone suppresses the expression of VEGF-A and VE-cadherin but the effect of exercise on these parameters is again aldosterone independent. Collectively, the data do not support any evidence for a meaningful combination of aldosterone blockade and high physical activity to protect the vasculature against hypertension-dependent stress.

The number of monocytes did not significantly change by either running wheel activity or spironolactone and subsequently the number of CD206^+^ monocytes adherent at the aortic endothelium or invagination into the vascular wall did not show strong differences, with the exception of a similar trend for a reduced number of cells at the abluminal sites after running activity or spironolactone treatment. These data suggest that spironolactone has minimal effects on white-blood cell-dependent inflammatory processes seen in SHRs.

Our study is limited in the way that we can not directly conclude from each of the individual adaptation to hypertension and high physical activity to the physiological consequence. However, in total this study shows that lack of translation from promising protective effects from rats to patients may be related to co-morbidities rather than to species differences. As mentioned in the discussion, many of the observed changes were found similar in humans in response to exercise.

In summary the current study reveals that protective effects of high physical activity on white blood cell counts and vascular adaptation in normotensive rats cannot simply be transferred into the complex interaction between circulating leukocytes and the vasculature in hypertensive rats. Furthermore, the data show that some of the most characteristic effects of spironolactone seen in sedentary rats, are reversed when combined with high physical activity in SHRs. Based on our study we conclude that the majority of adaptive processes specifcally seen in SHRs are independent from aldosterone effects and even overrun the protection seen with inhibition of aldosterone effects.

## Data Availability Statement

The original contributions presented in the study are included in the article/supplementary material, further inquiries can be directed to the corresponding author/s.

## Ethics Statement

The animal study was reviewed and approved by RP Gießen; V 54–19 c 20 15 h 01 GI 20/1 Nr. 76/2014 and GI 20/1 Nr.77/2014.

## Author Contributions

RS, AW, and SS performed experiments with animals. CT performed experiments with FACS. RS and K-DS concept. K-DS manuscript writing and preparation. RS and AW proof reading. All authors contributed to the article and approved the submitted version.

## Conflict of Interest

The authors declare that the research was conducted in the absence of any commercial or financial relationships that could be construed as a potential conflict of interest.

## Publisher's Note

All claims expressed in this article are solely those of the authors and do not necessarily represent those of their affiliated organizations, or those of the publisher, the editors and the reviewers. Any product that may be evaluated in this article, or claim that may be made by its manufacturer, is not guaranteed or endorsed by the publisher.
